# The Role of Host Cell DNA Methylation in the Immune Response to Bacterial Infection

**DOI:** 10.3389/fimmu.2021.696280

**Published:** 2021-07-29

**Authors:** Wanhai Qin, Brendon P. Scicluna, Tom van der Poll

**Affiliations:** ^1^Center of Experimental & Molecular Medicine, Amsterdam University Medical Centers, Academic Medical Center, University of Amsterdam, Amsterdam, Netherlands; ^2^Department of Clinical Epidemiology, Biostatistics and Bioinformatics, Amsterdam University Medical Centers, Academic Medical Center, University of Amsterdam, Amsterdam, Netherlands; ^3^Division of Infectious Diseases, Amsterdam University Medical Centers, Academic Medical Center, University of Amsterdam, Amsterdam, Netherlands

**Keywords:** DNA methylation, immune response, bacteria, infection, mechanism, review

## Abstract

Host cells undergo complex transcriptional reprogramming upon infection. Epigenetic changes play a key role in the immune response to bacteria, among which DNA modifications that include methylation have received much attention in recent years. The extent of DNA methylation is well known to regulate gene expression. Whilst historically DNA methylation was considered to be a stable epigenetic modification, accumulating evidence indicates that DNA methylation patterns can be altered rapidly upon exposure of cells to changing environments and pathogens. Furthermore, the action of proteins regulating DNA methylation, particularly DNA methyltransferases and ten-eleven translocation methylcytosine dioxygenases, may be modulated, at least in part, by bacteria. This review discusses the principles of DNA methylation, and recent insights about the regulation of host DNA methylation during bacterial infection.

## Introduction

DNA methylation refers to the addition of a methyl group to the DNA cytosine residues at the fifth carbon position (5mC), which is a common epigenetic mark in many eukaryotes and often found in the sequence context CpG (i.e., regions in the DNA where a cytosine nucleotide is followed by a guanine nucleotide along the 5’ to 3’ direction) (1). The methylation process is promoted by the DNA methyltransferases (DNMTs), of which DNMT3A and DNMT3B mediate *de novo* DNA methylation, establishing a pattern of methylation that is then sustained by the maintenance methyltransferase, DNMT1 (2). DNMT2 is not involved in DNA methylation, but rather mediates methylation of RNA (3), and therefore is further not discussed in this review. The process of DNA methylation can be reversed passively through cell division or actively catalyzed by ten-eleven translocation (TET) methylcytosine dioxygenases family proteins, and a subsequent nucleotide excision and repair process, called DNA demethylation (4). There are three members in the TET family, namely TET1, TET2 and TET3, all sharing a conserved catalytic domain in their C terminus (5). DNA methylation is generally associated with transcriptional silencing, although this paradigm has been challenged by recent studies showing that DNA methylation can both positively and negatively regulate gene expression depending on the position where it occurred (6).

Both innate and adaptive immune responses contribute to protection of the host against bacterial pathogens (7). The innate immune system functions as the first line of defense against invading pathogens and is composed of innate immune cells (including basophils, dendritic cells, eosinophils, Langerhans cells, mast cells, monocytes, macrophages, neutrophils and natural killer cells) and some stromal cells, such as epithelial cells that sense bacteria by their surface or endosomal pathogen recognition receptors (PRRs). Toll-like receptors (TLRs), RIG-I-like receptors, NOD-like receptors and C-type lectin receptors are among the large array of PPRs that are able to detect pathogens by recognizing microbial components known as pathogen-associated molecular patterns, among which lipopolysaccharide (LPS), flagellin and lipoteichoic acid (8, 9). Upon recognition of bacteria or bacterial components, innate immune cells initiate intracellular signaling cascades to induce functional changes and to elicit the production of immune effectors, such as cytokines, chemokines and antimicrobial peptides, that directly or indirectly contribute to host antibacterial defense and inflammatory responses. When bacterial pathogens evade host innate immunity, adaptive immune responses can contribute to defense mechanisms. T and B cells are dominant players in adaptive immunity, activated through presentation of bacterial antigens by antigen-presenting cells. Innate and adaptive immune responses do not act independently, but coordinated actions of these two systems are required for efficient elimination of bacterial invaders. Furthermore, in order to prevent collateral damage both innate and adaptive immune responses need to be tightly regulated at different levels (10). Modification of DNA methylation in host cells, induced by infectious agents, has been implicated in the induction and regulation of the immune response to bacteria.

DNA methylation has been considered to be relatively stable when compared with other epigenetic modifications, such as those involving histones, but recent findings have documented that DNA methylation can occur faster than previously thought, particularly when cells are exposed to changing environments, including contact with pathogens during infection (11). Importantly, accumulating evidence indicates that pathogens can alter DNA methylation and/or regulate the expression and function of DNA methylation modifiers such as TETs and DNMTs, resulting in altered expression of important host genes involved in immune responses (11). These alterations in DNA methylation or its related factors can either contribute to protective host immunity to eliminate pathogens or benefit pathogens to evade immune responses for persistence within the host. This review summarizes current understanding of the effects of DNA methylation on host immune responses and pathogen elimination during infection.

## DNA Methylation

**Figure 1:**
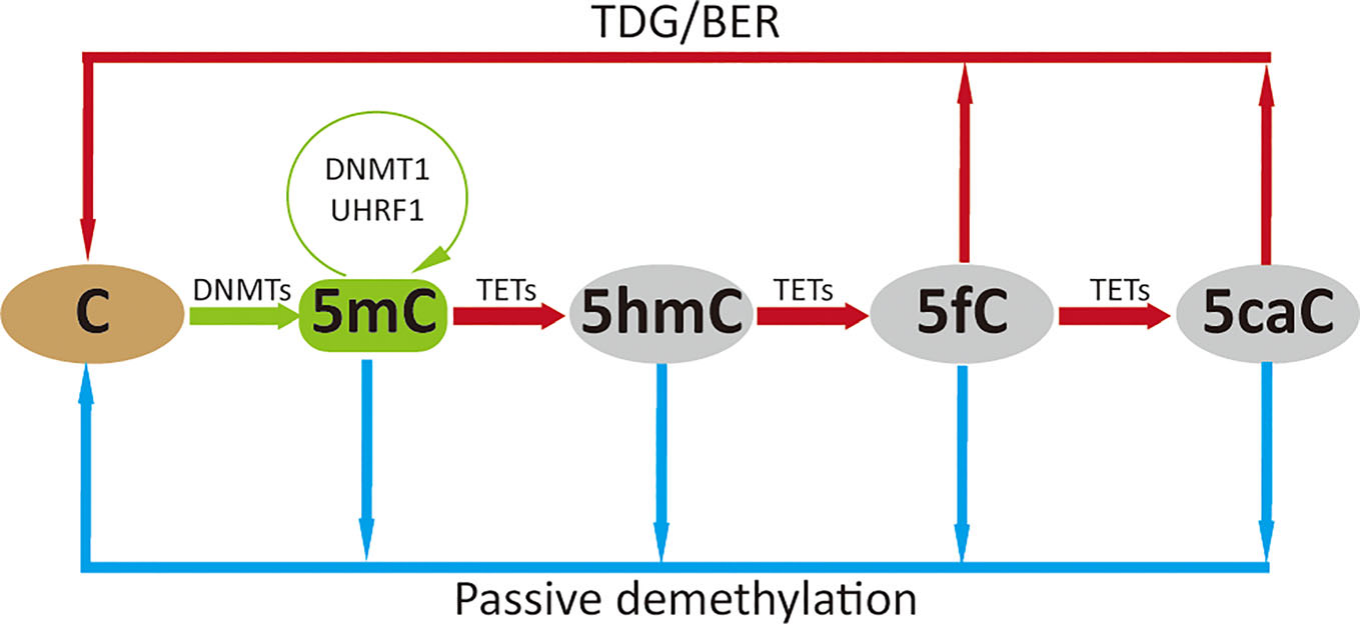
DNA methylation cycle. DNMTs catalyze the addition of a methyl group to the fifth carbon position of cytosine to generate methylated cytosine (5mC), which is maintained by DNMT1 (green arrow); 5mC is oxidized to 5-hydroxymethylcytosine (5hmC), which can be further oxidized to 5-formylcytosine (5fC) and 5-carboxylcytosine (5caC) by TETs. The higher oxidized cytosine bases 5fC and 5caC can then be converted back to their unmodified state directly by thymine DNA glycosylase (TDG) and subsequently base excision repair (BER) processing; these oxidative steps contribute to active demethylation (red arrow). Passive demethylation removes 5mC from all forms of methylcytosine due to absence or reduction in DNMT levels and function (blue arrow).

Two families of proteins directly contribute to the DNA methylation pathway: the DNMTs promote and maintain DNA methylation, while the TETs catalyze demethylation *via* multiple steps (**Figure 1**). DNA methylation is established by the *de novo* methyltransferases DNMT3A and DNMT3A with the help of catalytically inactive DNMT3L in mammals, whilst the maintenance of DNA methylation is mediated by DNMT1 and its obligate partner ubiquitin-like plant homeodomain and RING finger domain 1 (UHRF1), which preferentially recognizes hemimethylated CpGs during cell division (12).

Although DNA methylation is reported to be stable, DNA demethylation has been widely observed during development and activation of mammalian cells. Possible mechanisms underlying DNA demethylation have been reviewed by other researchers (13–16); we here only briefly introduce the broadly recognized passive and active routes. Passive demethylation occurs in the absence of the DNA methylation maintenance machinery (DNMT1/UHRF1) during DNA replication, which leads to dilution of 5mC, or removal of 5mC due to absence or reduction in DNMT levels and function (17). Active demethylation is mostly dependent on the oxidation of 5mC by TETs, that oxidize 5mC to 5-hydroxymethylcytosine (5hmC), which can be further oxidized to 5-formylcytosine (5fC) and 5-carboxylcytosine (5caC). These oxidized cytosine bases (5hmC/5fC/5caC) may facilitate DNA demethylation by impairing the binding and/or activity of enzymes regulating the maintenance methylation machinery (DNMT1/UHRF1) which impairs remethylation during DNA replication (13). The higher oxidized cytosine bases (5fC/5caC) can be efficiently excised by thymine DNA glycosylase (TDG), followed by the base-excision-repair (BER) pathway, which accounts for the major DNA demethylation mechanism. Interestingly, TETs might not decrease methylation levels, but specifically prevent aberrant methylation spreading into CpG islands (CGIs) (18), and DNMTs might also contribute to active DNA demethylation in conditions of low methyl group sources (19).

## Regulation of DNMTs

**Figure 2:**
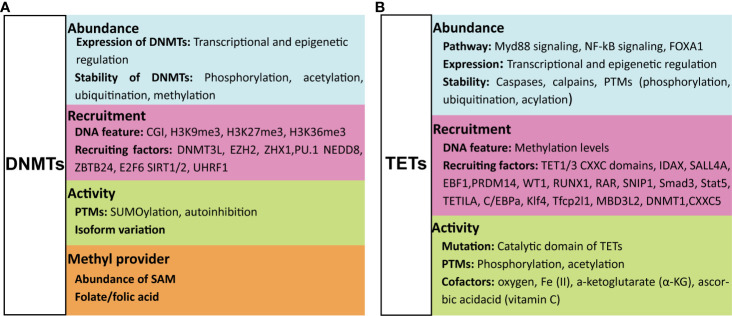
Factors that regulate the function of DNMTs and TETs. The function of DNMTs can be influenced at four levels: their abundance, their recruitment to DNA, their catalytic activity, and the methyl group source **(A)**. The function of TETs is regulated at three levels: their abundance, their recruitment to DNA and their catalytic activity **(B)**. For details see text. DNMTs, DNA methyltransferases; TETs, ten-eleven translocation methylcytosine dioxygenases; PTMs, post-translational modifications; CGI, CpG islands; SAM, S-adenosyl-L-methionine.

DNMT proteins are recruited to certain locations in the genome where they catalyze the transfer of methyl groups from S-adenosyl-L-methionine (SAM) to the C5 of cytosine to establish 5mC. During this process, the activity of DNMTs can be regulated at the following levels (**Figure 2**).

### First, by the Abundance of DNMTs

The expression and stability of DNMTs can be regulated by transcriptional regulation and post-translational modifications (PTMs), respectively. Numerous pathways have been shown to induce or inhibit expression of DNMTs, and the extent of their expression can be further regulated by multiple epigenetic regulatory mechanisms (20). Proteolytic degradation of DNMT proteins can be promoted or inhibited by PTMs. Acetylation and ubiquitination of DNMT1 either protect from or promote proteolytic degradation (21, 22). Phosphorylation of Ser143 stabilizes DNMT1 (23), whilst methylation of Lys142 and Lys1096 promotes its proteolytic degradation (24, 25).

### Second, Through the Function/Activity of DNMTs

DNA methylation by DNMTs is dependent on their catalytic activity, which is largely regulated by PTMs or isoform variation of DNMTs. SUMOylation of DNMT1 increases the catalytic activity of this enzyme on genomic DNA (26); SUMOylation of DNMT3A, however, abolishes its capacity to interact with histone deacetylases (HDACs) (27). DNMT1 is an auto-inhibitory protein that is activated upon binding to unmethylated cytosines (28, 29). The same auto-inhibitory characteristic was also found for DNMT3A, the activation of which is induced by histone H3 (30)**;** this is might be the reason why the histone H3 N-terminal tail with an unmethylated Lys4 (H3K4) is required for *de novo* DNA methylation (31). In addition, the activity of DNMTs can be affected by isoform variation (32, 33), and other regulatory proteins, such as the microprocessor component DROSHA that interacts with DNMT1 to ensure its full methyltransferase activity (34).

### Third, Through Recruitment of DNMTs to the Genome

To successfully perform DNA methylation, DNMTs are first recruited to the targeted DNA motif, and this recruitment is affected by both the features of the target DNA motif and factors that influence DNMT recruitment to the genome. DNMTs can be specifically recruited to DNA marked with unmethylated H3K4 *via* interacting with the ADD domain of DNMTs (35), while methylated H3K4 repulses the binding of *de novo* methyltransferases resulting in maintaining the hypomethylated state of CGIs (36). CGIs marked by H3K27me3 are more susceptible to *de novo* DNA methylation during differentiation and in disease states such as cancer (37, 38). Gene body enriched with H3K9me3 or H3K36 tri-methylation (H3K36me3) is also reported to be favorable for DNMT3B recruitment, leading to hypermethylation at these regions that functionally relate to gene transcription initiation, proper splicing and compact chromatin at active genes (37, 39, 40). The affinity of DNMT3A and DNMT3B for DNA can be further enhanced by DNMT3L through the formation of heterotetrametric complexes with either DNMT3A or DNMT3B, resulting in more efficient DNA methylation (41, 42). A large class of proteins, including polycomb group protein enhancer of zeste homolog 2 (EZH2) (43), Zinc-fingers and homeoboxes 1 (ZHX1) (44), ubiquitin-like protein modifier NEDD8 (45), zinc-finger protein ZBTB24, transcription factor E2F6 and PU.1, and Sirtuins 1 and 2 (SIRT1/2), were reported to recruit DNMTs to genes targeted for DNA methylation mediated gene silencing (46–49). The binding of DNMT1 to hemimethylated cytosines is selectively promoted by UHRF1 (50), but this binding is prevented by a DNA aptamer named Apt. #9 that competes with the hemiDNA for binding to DNMT1 (51). Besides protein molecules discussed above, some RNAs were also reported to affect the recruitment of DNMTs (52–54).

### Fourth, the Methyl Group Donors Determine the Direction of the DNA Methylation Pathway

SAM is the major source of methyl groups for DNA methylation. The addition of folate/folic acid to provide methyl groups was reported to maintain DNA methylation and/or prevent the loss of global DNA methylation in health and disease (55, 56). However, factors that lead to less SAM decreases the transfer of methyl groups to DNA and RNA (57). In the absence of SAM, DNMT3a and DNMT3b can exhibit DNA dehydryoxymethylase activity, by directly converting 5hmC and 5caC, but not 5fC, to unmodified cytosines (58, 59). In some cases, DNMT1 is able to mediate oxidation of cytosine with formaldehyde, forming 5hmC (60), which further can participate in the DNA methylation cycle.

## Regulation of TETs

The presence and catalytic activity of TETs are necessary for DNA demethylation, but their function is affected by multiple regulatory mechanisms that (amongst others) modulate substrate accessibility, enzymatic activity, expression levels and genomic targeting of TETs. Factors that are of importance for the regulation of activity of TETs are the following.

### First, the Abundance of TETs Can Be Regulated at Transcriptional and Post-Transcriptional Levels

The expression of TETs can be induced by multiple signaling pathways, such as hydrogen sulfide (61), Myd88 signaling (62), NF-kB signaling (63) and Forkhead box A1 (FOXA1) (64), and frequently regulated at transcriptional level. IDAX (also known as CXXC4) and lysine demethylase KDM2A (65) negatively regulate whilst transcription factors Oct4 and CEBPα positively regulate TET2 protein expression (66–68). TET3 can be negatively regulated by nuclear receptor TLX (69). More recently, TETs were shown to be regulated by epigenetic modifications involving long non-coding RNA’s or microRNA’s (70–73). The abundance of TETs can also be regulated at protein level. TETs can be directly cleaved by caspases (68) and calpains (74) or degraded through PTMs. For instance, all three TET proteins can be monoubiquitinated by the VprBP-DDB1-CUL4-ROC1 E3 ubiquitin ligase (CRL4VprBP) (75), whilst MAPK-mediated phosphorylation at Serine-99 of TET2 stabilizes this enzyme (76, 77). Moreover, the 14-3-3 proteins bind phosphorylated TET2 and protect Serine-99 phosphorylation (78). Other modifications like (de)acetylation of TETs have also been reported; for example, acetylation of TET2 by p300 stabilizes this enzyme by inhibiting ubiquitination (79), whilst deacetylation of TET2 by the deacetylase SIRT1 promotes its ubiquitination degradation as well as enhances its catalytic activity (80, 81).

### Second, the Binding of TETs to Genomic DNA Sequences Can Be Modulated

Similar to DNMTs, TET proteins also need to be recruited to the genome for implementing their functions. TET1 and TET3 can be recruited to genomic target sites through direct binding of their respective CXXC domains to DNA (82). This binding process can be influenced by several proteins. For instance, Lin28A recruits TET1 to common genomic loci to regulate DNA methylation and gene expression (83), thyroid hormone receptors stabilize the association of TET3 to chromatin depending on the catalytic activity of TET3 (84). In contrast to TET1 and TET3, TET2 is recruited to genomic DNA by a distinct CXXC domain-independent mechanism since TET2 does not have any discernable domains that bind directly to DNA. Indeed, numerous proteins have been discovered that promote or inhibit binding of TET2 to DNA. IDAX/CXXC4, originally encoded within an ancestral *TET2* gene but separated from *TET2* during evolution, recruits TET2 to DNA sequences containing unmethylated CpG dinucleotides located at promoters and CGIs in genomic DNA (68, 85). Other molecules such as Wilms tumor protein 1 (WT1) (86), early B-cell factor 1 (EBF1) (87), PRDM14 (88), RUNX1 (89), retinoic acid receptor (RAR) (90), SNIP1 (91), Smad3 and Stat5 (61), TET2 interacting long noncoding RNA (TETILA) (92) and transcription factors C/EBPa, Klf4, and Tfcp2l1 (93) can interact with TETs and enhance the recruitment of TETs to target loci. In addition, some proteins like Methyl-CpG binding domain protein 3-like 2 (MBD3L2) (94), DNMT1 (79), CXXC5 (95) and SALL4A (96) can further strengthen or stabilize the binding between TETs and methylated DNA targets. Besides factors modifying the recruitment of TETs, the character of target DNA sequences can also affect the binding of TETs. For example, low-methylated regions (LMRs) of CpG-poor distal regulatory regions that are occupied with DNA-binding factors are favorable for TET binding, thereby maintaining low methylation levels in these regions (97).

### Third, Dioxygenase Activity of TETs Is Tightly Regulated

The dioxygenase activity of TETs is largely dependent on their catalytic domain and any mutation or modification within this region is likely to lead to a change in their function. Enzymatic reactions mediated by TETs highly rely on the cofactors oxygen, Fe (II), and a-ketoglutarate (α-KG) (98). Therefore, any modification in the production or activity of these cofactors is expected to lead to a functional change of TETs. Mutations in the genes encoding the metabolic enzymes isocitrate dehydrogenases 1 and 2 (IDH1/2), succinate dehydrogenase, and fumarate hydratase, result in aberrant accumulation of metabolites such as 2-hydroxyglutarate (2-HG), succinate and fumarate, respectively, which act as competitors of α-KG to broadly inhibit the α-KG-dependent enzymatic activity of TETs (99–101). Hypoxia, such as frequently occurs in tumor tissues, leads to loss of TET activity (102). On the other hand, addition of ascorbic acid (vitamin C), which is needed to reduce the oxidized iron species, enhances the catalytic activity of TETs (103–105). Additionally, TETs activity has also suggested to be affected by PTMs. Acetylation enhances TET2 function (79) and phosphorylation of TET3 at the highly conserved Serine-1310 and -1379 residues within its catalytic domain by cyclin-dependent kinase 5 (cdk5) is required for its dioxygenase activity (106). Moreover, the phosphorylation of TETs can be suppressed *via* O-GlcNAcylation by the glycosyltransferase OGT (107).

## DNA Methylation and Gene Expression

### DNA Methylation, DNA Demethylation and Gene Expression

DNA methylation plays a critical role in the regulation of many cellular processes, including X chromosome inactivation, genomic imprinting, stem cell differentiation, chromosomal conformation, chromatin structure, developmental stages and transcriptional activation/repression of genes (108). DNA methylation in the genome is not uniformly distributed: both promoter and CGIs typically are hypomethylated, whereas the extent of methylation in gene bodies is higher than that in intergenic regions (2). While early studies suggested that DNA methylation represses gene expression, a growing body of evidence has indicated that DNA methylation has a dual role, both inhibitory and permissive, depending on the genomic region at which DNA methylation occurs (2). DNA methylation of CpGs at promoters and enhancers that usually remain unmethylated is mainly coupled with transcriptional silencing (108, 109), but DNA methylation at the gene body has been associated with enhanced gene transcription or elongation (39, 110). DNA methylation can also indirectly regulate gene expression by altering the chromatin accessibility for transcription factors or by recruiting repressive proteins with methyl-binding domains (111). For instance, DNA methylation changes the accessibility of B cell enhancers for transcription factors E2A and PU.1 and blocks the binding of transcription factor erythroblastosis 1 (ETS1) at Ets binding site during B cells development (112, 113). In addition, DNA methylation closely cooperates with other regulatory machineries to modify gene expression, especially with histone modifications, which can partially be mediated through methylcytosine-binding proteins, such as MECP2 or MBD2, that are capable of recruiting histone deacetylases or transcriptional repressors to methylated regions (111, 114). DNA demethylation, on the other hand, is normally positively correlated with gene transcription (13). However, the precise relationship between DNA (de)methylation and gene expression is complex and requires further investigation. For instance, it is reported that microbe-induced changes in the expression of some genes can occur prior to modification of DNA methylation at their sites (11, 115) and that elevated DNA methylation outside of gene promoters has been shown to facilitate gene transcription to a larger extent than promoter DNA methylation (116, 117).

### DNMT Related Gene Expression

DNMTs can repress gene expression by increasing DNA methylation at promoters and enhancers, resulting in reduced binding of transcriptional factors to these positions or inducing changes in the chromatin structure to make it less accessible for transcription (2, 111). For instance, DNMT3B mediated DNA methylation at the promoter regions of NF-κB responsive genes decreases NF-κB recruitment to the promoters, suppressing the expression of downstream genes (33). H3k6me3 selectively recruits DNMT3B to gene bodies of actively transcribed genes, thereby promoting DNA methylation and gene expression (37, 39, 110, 118). DNMTs can regulate gene expression not only *via* directly modifying DNA methylation, but also through mechanisms that are unrelated to DNA methylation but achieved by cooperating with other regulatory machineries. All three DNMTs (DNMT1, 3A and 3B) have been reported to repress gene transcription through interacting with HDACs independent of their catalytic activity (27, 119). DNMT3A-mediated DNA methylation increases HDAC9 transcription by repressing the inhibitory histone mark H3K27me3 at its distal promoter (116). DNMTs work together with polycomb group proteins for repression of their common target loci (43). The tricarboxylic acid cycle metabolites succinate and fumarate determine the catalytic activity of DNMTs; in turn, DNMT3B has been reported to modulate mitochondrial metabolism for maintaining articular cartilage homeostasis (120).

### TET Related Gene Expression

TETs regulate gene expression directly by demethylation, dependent on their catalytic activity, or indirectly through interaction with other regulatory mechanisms, mostly independent of their catalytic activity. All three TETs contribute to dynamic demethylation during development, activation and oncologic transformation, linked with wide transcription reprogramming in cells during these processes (5, 121). In recent years, more and more DNA methylation independent functions of TETs have been discovered, indicating that TETs closely work together with other epigenetic regulatory mechanisms in the setting of infection. TET2 and TET3 have been shown to inhibit proinflammatory cytokine expression by recruiting HDAC1/2 to the promoters of cytokine encoding genes during bacterial and viral infection, respectively (122–124). TET2 also mediated transcriptional repression by facilitating the recruitment of the polycomb Repressive Complex 2 to CpG dinucleotide-rich gene promoters (125). TET1 can be incorporated in the SIN3A co-repressor complex, resulting in transcriptional effects independent of 5hmC (126), and this might be the underlying mechanisms of TET1 mediated inhibition of *IL1B* transcription (127). The same mechanism applies to TET3 regulated inhibition of type I interferon production during viral infection or poly(I:C) stimulation (124). TET2 and TET3 facilitate OGT-dependent histone O-GlcNAcylation by interacting with the enzyme O-linked b-N-acetylglucosamine (O-GlcNAc) transferase (OGT) (128, 129). Beyond oxidation of methylated cytosine in DNA, TET2 has also been reported to promote mRNA oxidation during infection derived sepsis, thereby destabilizing target mRNA (130); TET2 can suppress expression of endogenous retroviruses through a similar mechanism (131).

## Modification of DNA Methylation Associated With Infection

**Figure 3:**
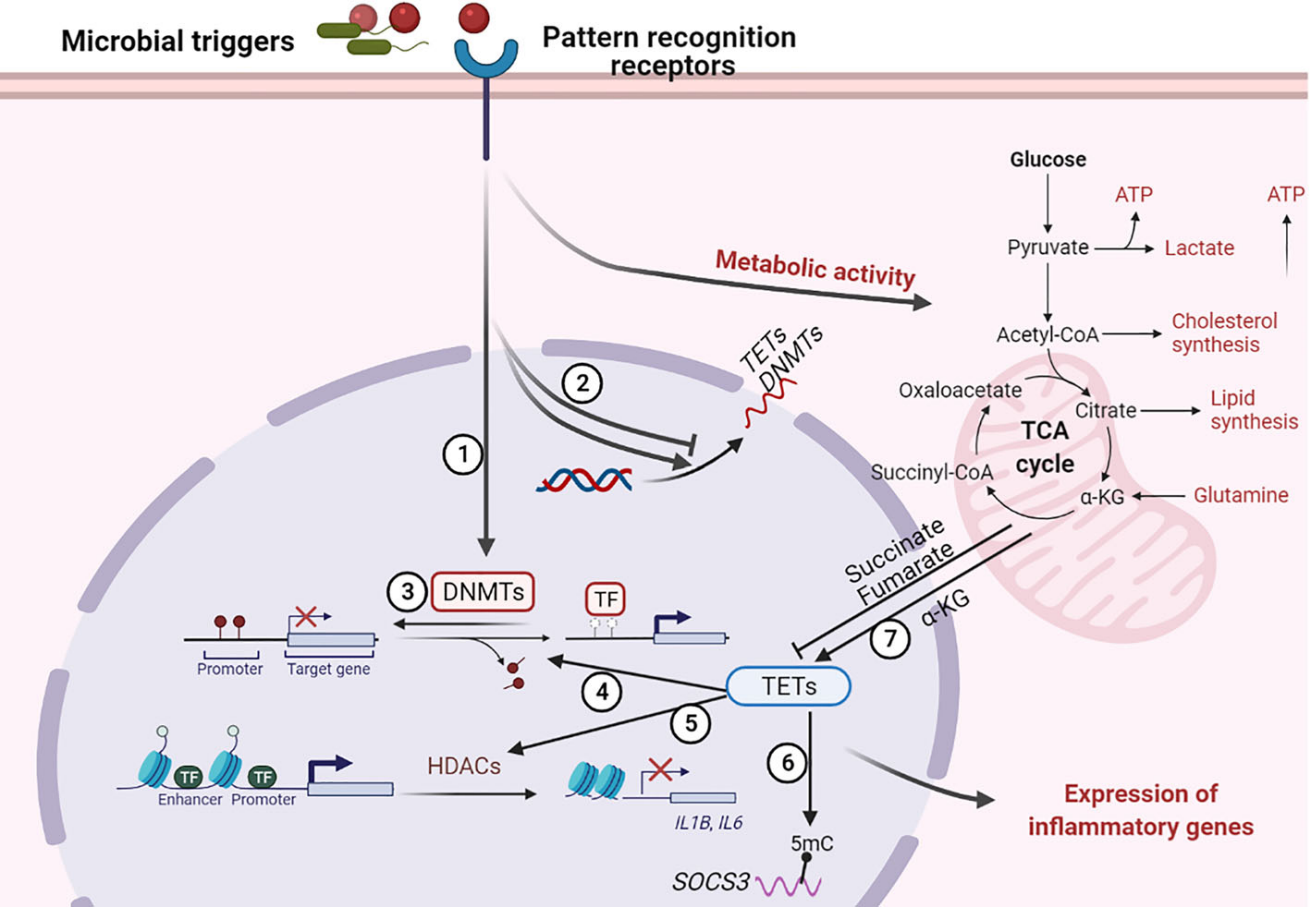
Regulation of host DNA methylation of immune responses during infection. Figure representing a general overview of how infection can affect DNA methylation. Note: not all infection modify DNA methylation; an overview of changes induced by specific pathogens is provided in the table. ① Infection induces DNA (de)methylation at target genes; ② Infection alters the transcription of DNA methylation modifiers TETs and DNMTs; ③ Loss of DNMTs promotes infection induced DNA demethylation at target genes; ④ TET proteins promote infection induced DNA demethylation at target genes; ⑤ TET proteins recruit HDACs for histone modification at *IL1B* and *IL6* promoters; ⑥ TET proteins oxidize 5-methylcytosine (5-mC) on SOCS3 messenger RNA (mRNA); ⑦ Infection alter metabolic products that regulate the activity of TET proteins. “arrow” symbol represents promotion, “bar-headed arrow” symbol represents inhibition. DNMTs, DNA methyltransferases; TETs, ten-eleven translocation methylcytosine dioxygenases; HDACs, Histone deacetylases; TFs, transcription factors; IL, interleukin; SOCS3, Suppressor of cytokine signaling 3; ATP, Adenosine triphosphate; α-KG, a-ketoglutarate.

The host response to an infection involves transcriptional changes in different types of immune cells, which can affect their function to either promote host defense against invading pathogens or benefit pathogen persistence. The transcriptional reprogramming during infection is highly regulated and epigenetic regulatory mechanisms are involved herein (132, 133) (**Figure 3**). Until recently, the extent of DNA methylation was thought to be stable and resistant to environmental stimulation. However, it is now well recognized that DNA methylation can be altered in a brief time frame in response to inflammation or infection and that these modifications in DNA methylation can influence immune cell responsiveness (11). Two possible mechanisms underlie infection induced alterations in DNA methylation: infection can directly alter DNA methylation by inducing or repressing DNA methylation enzymes (DNMTs and TETs), and/or indirectly through inflammatory mediators induced by the infection (134). Modification of host DNA methylation associated with bacterial infection and the consequent effects on immune responses were summarized in **Table 1** and detailed below.

**Table 1:** Modification of DNA methylation induced by bacteria and its effects on immune responses.

Bacteria	Effect on DNA methylation	Impact on immune response	References
**Gut microbiota**	Altered DNA methylation in IECs	Changed expression of genes related to immunity and metabolism in IECs	(135–137)
	Hypermethylation of *TLR4* in IECs	Suppressed response to LPS and commensal microbiota, maintaining intestinal homeostasis	(138, 139)
	Demethylation in IECs mediated by TET2/TET3	Maintained intestinal homeostasis and inhibition of acute inflammation in experimental colitis	(137, 140)
**Polymicrobial**	Altered DNA methylation in whole blood leukocytes	Changed gene expression in whole blood leukocytes of septic patients	(141, 142)
	Altered DNA methylation in monocytes	Increased IL-10 and IL-6 levels and organ dysfunction in septic patients	(143)
	Altered expression of DNMTs and TETs	Increased disease severity in septic patients or experimental septic mice	(130, 144, 145)
***Helicobacter pylori***	Aberrant DNA methylation in gastric mucosae caused by infection induced inflammation	Increased risk of gastric cancer	(134, 146–149)
	Aberrant DNMT activity in gastric tissues	Increased susceptibility to infection	(55, 150)
***Mycobacterium tuberculosis***	Altered DNA methylation in dendritic cells and macrophages *in vitro* and *in vivo*	Altered transcription of genes involved in immune response	(11, 151, 152)
	Aberrant DNA methylation in monocytes	Increased disease severity	(153–155)
	Demethylation at the promoter region of *Nlrp3* in macrophages	Increased NLRP3 inflammasome activation and downstream release of IL-1β and IL-18	(156)
	Aberrant methylation at the *TLR2* promoter in human blood leukocytes	Negatively regulated *TLR2* expression; increased bacterial burden and disease severity	(154)
***Escherichia coli***	Aberrant DNA methylation by altered DNMT activity in T cells	Dysregulation of immune responses to bacterial infection induced lung injury	(157, 158)
	Increased DNMT1 activity in uroepithelial cells	Downregulation of *CDKN2A* (tumor suppressor gene) and increased risk of bladder cancer consequently	(159, 160)
	Decreased DNMT3A activity in porcine mammary epithelial cells	Enhanced immune response	(161)
	Downregulation of *TET1* in THP1 macrophages	Reduced NF-κB signaling pathway and inhibition of macrophage M1 polarization	(162)
***Salmonella***	Altered DNA methylation in chicken cecum and blood leukocytes	Changed expression of immune and metabolic genes	(163, 164)
	Enhanced DNA methylation at the promoters of *TLR4*, *TLR21* and *TLR2-1* in chicken blood leukocytes	Reduced MyD88 signaling and increased susceptibility to *Salmonella enterica*	(165, 166)
***Pseudomonas aeruginosa***	Altered DNA methylation at *NODAL* in bronchial epithelial cells	Changed airway homeostasis	(167)
	Aberrant function of DNMT3B	Increased susceptibility to infection	(168, 169)
***Methicillin-resistant Staphylococcus aureus***	Reduced *DNMT3A* in macrophage and neutrophils	Reduced IL-10 production and increased inflammatory responses in patients; Increased susceptibility and mortality in murine models	(170)
	Modified DNA methylation signatures in circulating immune cells	Increased disease severity in patients	(171)
***Campylobacter rectus***	Hypermethylation of *Igf2* in mouse placenta	Down-regulation of *Igf2* and aberrant placental growth	(172)
***Porphyromonas gingivalis***	Decreased *DNMT1* expression in gingival epithelial cells	Increased antibacterial responses by promoting β-defensin 2 and CC chemokine ligand 20 expression	(173)
***Anaplasma phagocytophilum***	DNA hypermethylation in neutrophils potentially by promoting *DNMT3A* expression	Reduced neutrophil antibacterial functions	(174)
**Bacterial products**	**Effect on DNA methylation**	**Impact on immune response**	**References**
**LPS**	Aberrant DNA methylation at *TLR*s, inflammatory cytokines (*IL6*, *TNF*)	Dysregulation of cellular responses to LPS stimulation	(175–178)
	Increased DNMT1 activity in macrophages	Enhanced inflammatory responses by hypermethylation of anti-inflammatory factors such as KLF4, miR-145 and SOCS3	(178–180)
	Downregulation of *TET1* in macrophages	Inhibition of NF-κB signaling and decreased inflammatory responses	(162)
	Increased *Tet2* expression in myeloid cells	Decreased IL-6 production and reduced inflammation *in vivo*	(63, 122)
***Staphylococcal* enterotoxin B**	Modified DNA methylation of some genes with important roles in immunity in nasal polyp explants	Potentially altered immune responses related to T-cell maturation/activation	(181)
**Peptidoglycan and lipoteichoic acid**	Suppressed DNMT activity and hypomethylation of global DNA	Enhanced inflammatory responses	(182)
**Rv2966c from *Mycobacterium tuberculosis;* Mhy1, Mhy2, and Mhy3 produced by *Mycoplasma hyorhinis***	Hypermethylation of host genes by acting as DNA methyltransferase	Interference with host immune response	(183–185, 212)
**Extracellular vesicles secreted by *P. aeruginosa***	Modified DNA methylation at enhancers of immune-related genes in human lung macrophages	Abnormal innate immune response	(203)
**Bacterial metabolite folate**	Increased DNMT activity with altered DNA methylation in host cells	Unknown	(186)

IECs, intestinal epithelial cells; TLR, Toll-like receptors; LPS, lipopolysaccharide; DNMT, DNA methyltransferase; TET, ten-eleven translocation methylcytosine; KLF4, Krüppel-like factor 4; SOCS3, Suppressor of cytokine signaling 3; IL, interleukin; TNF, tumor necrosis factor.

### Gut Microbiota and Intestinal Pathogens

Commensal bacteria contribute to the maintenance of intestinal symbiosis by shaping host gene expression *via* epigenetic modification (187). Gut microbiota-dependent and -independent processes act together to form the postnatal development of the transcriptome and DNA methylation signatures of intestinal epithelial cells (IECs) early after birth. The formation of microbiota related “functional” methylation sites might impact long-term gene expression signatures in IECs (135, 136). Furthermore, some intestinal genes, related to innate immunity, phagocytosis, endothelial homeostasis and tissue metabolism are influenced by microbiota through DNA methylation (136). For instance, exposure of colonic epithelial cells to commensal bacteria results in Toll-like receptor (*TLR*)*4* gene hypermethylation and transcriptional downregulation, thereby suppressing responsiveness to LPS (138, 139). More importantly, TET2/3 in IECs contribute to enhanced demethylation induced by microbiota under homeostasis and during acute inflammation (137). Besides IECs, the development and function of immune cells at nonmucosal sites, such as the bone marrow, peripheral lymph nodes and spleen, are also suggested to be regulated by microbiota *via* DNA methylation (188). On the other hand, TET2 deficiency in hematopoietic cells can lead to a microbiota-dependent impairment of gut barrier (140).

Many intestinal pathogenic bacteria have been suggested to cause aberrant DNA methylation in host cells. In this context. *Helicobacter (H.) pylori* is one of the most investigated enteric pathogens. *H. pylori* is able to change DNA methylation directly. High levels of aberrant DNA methylation in *H. pylori*–infected gastric mucosae have been associated with gastric cancer risk (146). Indeed, several tumor suppressing genes were found downregulated in gastric mucosae through *H. pylori*–infection induced hypermethylation. DNA methylation at the promoter region of trefoil factors, which regulate mucosal repair and suppress tumor formation in the stomach, was found increased early after *H. pylori* infection and throughout gastric tumor progression (189). Similarly, hypermethylation of DNA repair protein O6-methylguanine DNA methyltransferase (MGMT) and reduced levels of MGMT were common in the gastric epithelium of *H. pylori* infected patients, increasing mutagenesis in *H. pylori*-infected gastric mucosa (190). Other important genes like *CX32* and *CX43* were also repressed by *H. pylori* induced hypermethylation (191). DNA hypermethylation in the context of *H. pylori* infection was partially reversible after eradication of this bacterium or administration of a DNA demethylating agent, 5-aza-2-deoxycytidine, resulting in decreased the incidence of gastric cancers induced by *H. pylori* infection (190, 192). Single nucleotide polymorphisms in *DNMT1* were reported to be genotypic markers for predicting genetic susceptibility to *H. pylori* infection (150), whilst the addition of folic acid to promote the activity of DNMTs was able to counteract *H. pylori* induced DNA demethylation (55), suggesting a direct role for methylation related factors herein. More recent evidence suggests that *H. pylori* induced inflammatory responses rather than the bacteria itself cause aberrant DNA methylation in the gastric mucosa (147). DNA hypermethylation induced by *H. pylori* infection was associated with down-regulation of genes involved in cell cycle progression control and DNA repair, thereby increasing the risk for gastric cancer (148). Mechanisms implicated in DNA hypermethylation during *H. pylori* infection include inflammation associated with the infection (134, 149) and altered expression or activity of DNA methylation related enzymes (62); as an example, IL-1β is able to induce *TET2* expression in macrophages *via* IL-1R-Myd88 signaling (62).

### Polymicrobial Infection and Sepsis

Sepsis is defined as life-threatening organ dysfunction resulting from a dysregulated host response to infection (193) and one of the leading causes of death globally (194). Sepsis is associated with changes in DNA methylation patterns in blood leukocytes of critically ill patients, and the majority of the differentially methylated region-associated genes were differentially expressed (141). Functional analysis showed that these sepsis related alterations in DNA methylation involved inflammatory pathways participating in both the innate and adaptive immune response, as well as in cell adhesion and cell junctions (141, 195). Likewise, the altered DNA methylation profiles in monocytes of septic patients correlated with increased IL-10 and IL-6 levels, as well as with organ dysfunction (143). Analysis of the CpG methylation status in blood cells of neonates with sepsis showed differential methylation of several CpGs located in functionally important genes including a group of *PCDHB* genes that play vital roles in leukocyte cell adhesion and the Wnt signaling pathway when compared to health (142). Another investigation indicated that the DNA methylation pattern of CpG sites in the promoter region of the calcitonin-related polypeptide α (*CALCA*) gene might be used as an epigenetic biomarker for bacterial sepsis in preterm newborns (196). Sepsis associated DNA methylation signatures in either specific genes or at genome-wide level have potential as diagnostic tools for predicting sepsis outcome or distinguishing sepsis subtypes. For instance, methylation of the NF-κB binding site in the Aquaporin5 (*AQP5*) promoter diminishes the binding of NF-κB and increased the expression of *AQP5* in blood cells of septic patients is associated with substantially greater 30-day mortality (197). Similarly, DNA methylation signatures in critically ill adults can distinguish septic and nonseptic patients, and can associate with clinical traits including severity of illness, need for vasopressors, and length of stay (141). These changes in DNA methylation likely at least in part are caused by sepsis-induced changes in the levels of enzymes mediating DNA methylation, as indicated by decreased DNMT1 and increased TET2 mRNA levels in blood leukocytes of sepsis patients (144). However, *de novo* DNMT mRNAs (*DNMT3A* and *DNMT3B*) in extracellular vesicles in blood were much higher than in healthy controls and strongly correlated with disease severity; DNMT mRNA levels were higher in septic shock patients than in sepsis patients without shock (145). In sepsis models, the inhibition of DNA methyltransferases by Decitabine attenuated NF-κB activation, downregulated inflammatory cytokine levels, inhibited the progression of sepsis and improved survival in mice with severe sepsis induced by cecal ligation and puncture (198). The presence of TET2 impaired survival in mice with sepsis by promoting emergency myelopoiesis and a cytokine storm through oxidation of 5-mC in Socs3 mRNA resulting in destabilization of this mRNA (130). Collectively, DNA methylation could be a potential diagnostic tool or biomarker for sepsis, and manipulation of DNA methylation enzymes might be a novel strategy in the treatment of sepsis.

### Specific Pathogens

#### Mycobacterium tuberculosis

*Mycobacterium tuberculosis* (MTB) infection has been reported to change DNA methylation at global level and at specific target CpGs both *in vivo* and *in vitro*. An *in vitro* study showed that MTB infection can lead to rapid changes in DNA methylation in non-proliferating cells, in parallel with the transcriptional response (11). Altered DNA methylation in macrophages was predominantly found at non-CpG dinucleotide sites during MTB infection (151), and the mycobacterial protein Rv2966c might be responsible for this type of DNA methylation change (183). Macrophages isolated from MTB infected patients also showed altered DNA methylation profiles of the promoter sequences of many cytokines and their receptors (152). For instance, demethylation at the promoter region of *NLRP3* by MTB infection activates the NLRP3 inflammasome and increases IL-1β and IL-18 release (156). Peripheral blood mononuclear cells from TB patients are characterized by DNA hyper-methylation of genes critical to mycobacterial immunity resulting in decreased mycobacteria-specific and non-specific immune responsiveness (153). Aberrant methylation of certain CpG sites over the *TLR2* promoter negatively regulated *TLR2* expression in NK cells/monocytes of patients with active pulmonary TB and correlated with the bacterial burden and disease severity (154); likewise, increased DNA methylation in monocytes from tuberculosis patients was suggested to reflect disease severity (155). Collectively, these results suggest that DNA methylation profiles of leukocyte subsets might be used as clinically prognostic tools for TB.

#### Escherichia coli

*Escherichia (E.) coli* is a Gram-negative and common causative pathogen in gastroenteritis, urinary tract infection, neonatal meningitis, hemorrhagic colitis, peritonitis and pneumonia. Several studies have documented modifications of DNA methylation in host cells during *E.coli* infection. DNA methylation within the promoters of a core set of CD4^+^ T-cell pathway genes attenuated neonatal immune responses to pneumonia-induced injury (157). Yet, DNMT inhibition by 5-aza-2-deoxycytidine (DAC) augmented the number and function of regulatory T cells thereby accelerating the repair of experimental lung injury (158), suggesting that the altered DNA methylation might be caused by the changes in the abundance or activity of regulatory enzymes during *E.coli* infection. Moreover, *E. coli* induced alterations in DNA methylation are frequently accompanied by changes in the expression of genes encoding proteins that are required for controlling bacterial infection. Uropathogenic *E. coli* infection induces *de novo* methyltransferase activity and *DNMT1* expression causing increased methylation of *CDKN2A* exon 1 and downregulation of this tumor suppressor gene in uroepithelial cells, which may increase the risk of bladder cancer (159, 160). However, downregulation of *de novo* methyltransferase *DNMT3A* by *E. coli* was accompanied by hypomethylation of some immune response genes in porcine mammary epithelial cells (161). Additionally, knockdown of *TET1* in THP1 macrophages downregulated the activity of the NF-κB signaling pathway activated by *E. coli*, thus inhibiting macrophage M1 polarization (162). Avian pathogenic *E. coli* infection led to changes of DNA methylation at gene body regions in the spleen, which negatively correlated with the expression of genes involved in the host inflammatory response and other networks and pathways related to injury/survival (199).

#### Salmonella

*Salmonella* is the most frequently detected causative agent in foodborne outbreaks worldwide. *Salmonella (S.) typhimurium* and *S. enteritidis* are the most common serotypes associated with foodborne diseases (200). The domestic chicken is an important host of *S. enterica*, and some studies showed that *S. enterica* infection alters DNA methylation in immune and metabolism related genes in chicken cecum and blood leukocytes (163, 164). Furthermore, enhanced DNA methylation levels at the promoters of *Tlr4*, *Tlr21* and *Tlr2-1* of blood leukocytes is related to reduced expression of genes in the MyD88 signaling pathway and increased susceptibility to *S. enterica* infection (165, 166). Notably, although *Salmonella* is an important pathogen in humans, knowledge of its capacity to modify DNA methylation in human cells is lacking.

#### Pseudomonas aeruginosa

*P. aeruginosa* is one of the main causative pathogens in hospital-acquired pneumonia and chronic airway infection associated with cystic fibrosis (201). Bronchial epithelial cells (BECs) are activated by and required for host defense against *P. aeruginosa* infection (202). Recently *P. aeruginosa* was shown to inhibit *NODAL* expression in BECs through methylation modification of its promoter. Nodal is vital for regulating proliferation of BECs and BEC-induced differentiation of T helper (Th) cells from Th1 to Th2 and Th17, thus regulating the immunological balance of the airway microenvironment (167). DNA methylation in human lung macrophages can be modified by *P. aeruginosa* secreted extracellular vesicles; DNA methylation modifications particularly occurred at distal DNA regulatory elements, including enhancer regions and DNase hypersensitive sites, and some CpGs associated with cytokines such as *CSF3* displayed strong negative correlations between DNA methylation and gene expression (203). DNA methylation enzymes are important for regulating host immune responses against this bacterium infection, as indicated by the association between genetic variants of *DNMT3B* and *P. aeruginosa* infection in children (168). We recently identified a role for DNMT3B in bronchial epithelial cells during *P. aeruginosa* pneumonia (169). DNMT3B deficient human bronchial epithelial cells produced more CXCL1 and related chemokines than control cells when stimulated with *P. aeruginosa*. Mechanistically, DNMT3B deficiency reduced DNA methylation at exon 1 of *CXCL1* and increased NF-ĸB p65 binding to the *CXCL1* promoter. These *in vitro* findings were corroborated by studies in mice with bronchial epithelial Dntm3b deficiency infected with viable *P. aeruginosa via* the airways, which showed increased *Cxcl1* expression in bronchial epithelium and CXCL1 protein release together with enhanced neutrophil recruitment and accelerated bacterial clearance. Additional studies using purified flagellin (an important virulent factor expressed by *Pseudomonas*) and a flagellin-deficient *P. aeruginosa* strain demonstrated that bronchial epithelial DNMT3b impaired host defense during *Pseudomonas* induced pneumonia at least in part by diminishing mucosal responses to flagellin (169). In separate investigations we showed that the DNA methylation eraser TET2 maintains epithelium barrier function during acute *P. aeruginosa* infection in mice (204).

#### Burkholderia pseudomallei

*B. pseudomallei* is an intracellular Gram-negative pathogen causing melioidosis, a common cause of sepsis in Southeast Asia and Australia. *B. pseudomallei* induced changes in DNA methylation of human macrophage-like U937 cells *in vitro*, particularly in the vicinity of genes involved in inflammatory responses, intracellular signaling and apoptosis (205).

#### Methicillin-Resistant Staphylococcus aureus (MRSA)

MRSA infection significantly decreased *DNMT3A* in blood leukocytes *in vivo* and in macrophage and neutrophils *in vitro*. *DNMT3A* knockdown increased *S. aureus* induced IL-10 production by macrophages *in vitro* and pretreatment with DAC increased mortality in a *S. aureus* murine sepsis model. However, a *DNMT3A* polymorphism increased the capacity to resolve MRSA bacteremia, potentially by reducing IL-10 production though a DNA methylation dependent mechanism (170). Indeed, persistent and resolving MRSA bacteremia were associated with different DNA methylation signatures in circulating immune cells of patients, particularly in neutrophils, and this distinct DNA methylation patterns were able to predict persistent MRSA bacteremia (171).

#### Campylobacter rectus

Placental and fetal infection with *C. rectus* in mice caused hypermethylation in the promoter region of *Igf2* in the placenta, resulting in down-regulation of *Igf2*, which affects the growth of the fetus by controlling both the placental supply of, and the genetic demand for, maternal nutrients to the fetus (172).

#### Porphyromonas gingivalis

*P. gingivalis*, the major pathogen in chronic periodontits, modifies *DNMT1* expression and changes methylation at the promoter region of several genes implicated in the innate immune response against bacteria and during tissue remodeling, whilst the DNMTs inhibitor DAC restores the expression of these genes in infected gingival epithelial cells (173).

#### Anaplasma phagocytophilum

*A. phagocytophilum* is a Gram-negative bacterium with a strong tropism for neutrophils that causes human granulocytic anaplasmosis, a zoonosis transmitted by ticks. *A. phagocytophilum* infection induces genome-wide hypermethylation in neutrophils potentially by promoting *DNMT3A* expression (174). Furthermore, inhibition of DNMTs by 5-azacytidine resulted in a partially recovery of neutrophil antibacterial functions and decreased bacterial growth (174).

### Bacterial Products

DNA methylation of immune cells can affect their responsiveness to microbial products, as illustrated by strong correlations between DNA methylation in human peripheral blood mononuclear cells and IL-6 production elicited by various TLR agonists (206). LPS is one of the major virulence factors of Gram-negative bacteria and the most used molecule for studying mechanisms underlying cellular immune responses. Recent evidence has indicated that changes in DNA methylation regulate LPS-induced immune responses and that modifying DNMT activity influences cellular responses to LPS (175). One way by which DNA methylation might influence LPS responsiveness is by affecting the expression of TLR4, the LPS receptor, as has been documented in intestinal epithelial cells (207). However, the most frequently reported mechanisms by which DNA methylation regulates LPS induced responses are associated with the function of DNA methylation modifiers. Increasing the methyl donor for DNA methylation by adding the *S*-adenosylmethionine (SAM) precursor methionine attenuated LPS-induced inflammatory responses in macrophages, whilst the DNMTs inhibitor DAC partially suppressed inflammatory responses induced by LPS in macrophages and other cell types (208, 209). Furthermore, DAC reduced lung inflammation and injury by inhibiting M1 macrophage activation *in vivo* (210). DNMTs were altered in bovine endometrial cells and microglia upon LPS stimulation and the expression of some inflammatory cytokines such as IL-1β, IL-6 and IL-8 were negatively regulated by methylation at their promoters (176, 177). Similarly, DNMT3B was reported to inhibit pro-inflammatory cytokine production by hypermethylation at their promoters or by downregulation of PPARγ expression (33, 211). Conversely, DNMTs mediated hypermethylation at promoters of anti-inflammatory factors, such as *SOCS1, KLF4* and miR-145 – and as a consequence thereof – their downregulation, exacerbates inflammatory responses either *in vivo* or *in vitro* (178–180). The role of TET proteins in LPS induced activation of immune cells was intensively studied, revealing both inhibitory and stimulatory functions. TET1 is able to interfere with the NF-κB signaling pathway and knockdown of *TET1* resulted in decreased production of proinflammatory markers by LPS/IFN-γ-induced M1 macrophages (162). TET2 functions downstream of the NF-κB signaling pathway by recruiting HDACs to the *IL6* promoter resulting in reduced *IL6* expression in macrophages and attenuation of inflammatory responses in murine endotoxemia model (63, 122). Besides LPS, there are few other bacterial compounds reported to affect DNA methylation in host cells. *Staphylococcus aureus* enterotoxin B altered the DNA methylation pattern in nasal polyp explants, most notably in *IKBKB* and *STAT5B*, genes encoding proteins with important roles in immunity (181). Likewise, peptidoglycan and lipoteichoic acid from this bacterium are able to suppress DNMT activity, resulting in enhanced inflammatory responses in bovine mammary epithelial cells (182). While the majority of bacterial compounds alter host DNA methylation by modifying the expression and activity of DNA methylation enzymes, mycobacterial protein Rv2966c by itself acts as a DNA methyltransferase that binds to host specific DNA sequences and methylates cytosines predominantly in a non-CpG context (183). Likewise, the swine pneumonia pathogen *Mycoplasma hyorhinis* produces Mhy1, Mhy2 and Mhy3, which can serve as mammalian DNMTs able to modify host DNA methylation (184, 185, 212). Besides bacterial components, bacterial metabolites might also affect host cell DNA methylation after uptake by these cells. For instance, folate produced by the commensal bacteria *Bifidobacterium* and *Lactobacillus* contributes to the generation of SAM resulting in increased DNMT activity and altered DNA methylation in host cells (186).

## Conclusion and Perspectives

Bacterial infection can alter the DNA methylation pattern of host cells, which may represent a strategy of pathogens to modify host gene expression to avoid clearance and facilitate colonization (213, 214). Changes in DNA methylation may also contribute to short-term memory in innate immune cells (215). Most of our current understanding of DNA methylation is derived from research fields outside infection immunity, in particular cancer and developmental immunology. Whilst awareness of the crucial role of DNA methylation and the proteins involved herein in regulating host immune defense against bacterial infection has increased, much remains to be learned about the mechanisms by which bacterial infection alters host DNA methylation and how this interferes with immune responses. Additionally, compared to a broad spectrum of bacteria that can modify host DNA methylation, thus far only few bacterial components or products have been reported to alter host DNA methylation, through mechanisms that are incompletely understood. Therefore, further research is warranted to reveal which bacterial effectors and mechanisms are involved in modification of host DNA methylation in bacterial infection. Expanding our knowledge of the role of variations in the methylation of DNA in host immune cells may not only enhance our understanding of host defense and the pathogenesis of bacterial infection, but also may provide clues for the development of novel therapeutics.

## Author Contributions

WQ and TP wrote the first draft of the article, with subsequent input from BC. All authors contributed to the article and approved the submitted version.

## Funding

WQ is supported by a Scholarship from the Chinese Scholarship Council (CSC).

## Conflict of Interest

The authors declare that the research was conducted in the absence of any commercial or financial relationships that could be construed as a potential conflict of interest.

## Publisher’s Note

All claims expressed in this article are solely those of the authors and do not necessarily represent those of their affiliated organizations, or those of the publisher, the editors and the reviewers. Any product that may be evaluated in this article, or claim that may be made by its manufacturer, is not guaranteed or endorsed by the publisher.
